# Type III Secretion System of Beneficial Rhizobacteria *Pseudomonas simiae* WCS417 and *Pseudomonas defensor* WCS374

**DOI:** 10.3389/fmicb.2019.01631

**Published:** 2019-07-16

**Authors:** Ioannis A. Stringlis, Christos Zamioudis, Roeland L. Berendsen, Peter A. H. M. Bakker, Corné M. J. Pieterse

**Affiliations:** Plant–Microbe Interactions, Department of Biology, Science4Life, Utrecht University, Utrecht, Netherlands

**Keywords:** beneficial rhizobacteria, type III secretion system, effectors, induced systemic resistance, rhizosphere

## Abstract

Plants roots host myriads of microbes, some of which enhance the defense potential of plants by activating a broad-spectrum immune response in leaves, known as induced systemic resistance (ISR). Nevertheless, establishment of this mutualistic interaction requires active suppression of local root immune responses to allow successful colonization. To facilitate host colonization, phytopathogenic bacteria secrete immune-suppressive effectors into host cells via the type III secretion system (T3SS). Previously, we searched the genomes of the ISR-inducing rhizobacteria *Pseudomonas simiae* WCS417 and *Pseudomonas defensor* WCS374 for the presence of a T3SS and identified the components for a T3SS in the genomes of WCS417 and WCS374. By performing a phylogenetic and gene cluster alignment analysis we show that the T3SS of WCS417 and WCS374 are grouped in a clade that is enriched for beneficial rhizobacteria. We also found sequences of putative novel effectors in their genomes, which may facilitate future research on the role of T3SS effectors in plant-beneficial microbe interactions in the rhizosphere.

## Introduction

Plants roots secrete significant amounts of carbon-rich compounds into the soil surrounding their roots, known as the rhizosphere, therewith shaping the microbial composition on their root system (Berendsen et al., 2012). In turn, these root microbiota can influence plant fitness and longevity either in a negative or a positive manner (Zamioudis and Pieterse, 2012). Beneficial members of the root microbiome provide the plant with important services, such as growth promotion, water and nutrient uptake, and protection against pathogens. *Pseudomonas* spp. bacteria are among the beneficial microbes that are strongly enriched in the rhizosphere in comparison to the bulk soil (Lugtenberg et al., 2001; Bakker et al., 2013). Members of this genus have been found to promote growth and nutrient uptake of their hosts, to compete with pathogens in the rhizosphere via antibiosis, and to trigger a systemic form of plant immunity, called induced systemic resistance (ISR) (Lugtenberg and Kamilova, 2009; Pieterse et al., 2014). To provide these benefits to their hosts, beneficial microbes need to colonize host roots, thereby outcompeting other microbes that aim to colonize the same niches and use the same carbon sources (Raaijmakers et al., 1995; Venturi and Keel, 2016). Like pathogenic *Pseudomonas* spp., beneficial *Pseudomonas* spp. need to cope with plant defense mechanisms in order to efficiently colonize their host (Pieterse et al., 2012; Zamioudis and Pieterse, 2012). For that, effective interference with local host immune responses is a prerequisite.

During coevolution with their hosts, animal- and plant-pathogenic *Pseudomonas* spp. have developed mechanisms that allow them to suppress or evade host defense responses and overcome basal immune responses (Jones and Dangl, 2006; Bardoel et al., 2011; Pel and Pieterse, 2013). The type III secretion system (T3SS) that delivers immune-suppressive effector molecules into the host cell, emerged as a conserved mechanism in Gram-negative bacteria employed for effective colonization of their host (Alfano and Collmer, 2004; Deslandes and Rivas, 2012). The T3SS is a protein secretion nanomachine composed of approximately 30 proteins. Electron microscopy has elucidated the three-dimensional structure of the T3SS, a needle complex consisting of a base that is composed of several rings and is anchored in the bacterial membrane, and a needle extension that is projected from the bacterial surface (Galán et al., 2014). Bacterial effector proteins that need to be secreted pass through the needle to become directly injected into the host cell. The proteins of the base and the ring structures that form the basal body of the secretion apparatus are conserved among different bacteria (Tampakaki et al., 2004) and can thus be easily identified in the genomes of Gram-negative bacteria of interest. Although the effector molecules that are secreted via the T3SS are not conserved, they contain molecular features that allows their identification in genome searches. Many effector-encoding genes from the bacterial leaf pathogen *Pseudomonas syringae* are characterized by the existence of a sequence in their promoter region, known as the “hrp box.” This motif is recognized by HrpL, a transcription (sigma) factor that regulates the expression of genes in the hrp operon (Chatterjee et al., 2002). Additional features reside in the N-terminal region of the effector protein sequence, such as the abundance of serine and polar amino acid residues, abundance of acidic amino acid residues in the first 12 amino acids, and an aliphatic amino acid in position 3 or 4 (Guttman et al., 2002; Petnicki-Ocwieja et al., 2002).

The existence of T3SS is not restricted to pathogenic bacteria. Also mutualistic root-associated bacteria, such as rhizobia and non-symbiotic plant growth-promoting rhizobacteria (PGPR) have been shown to possess a functional T3SS (Zamioudis and Pieterse, 2012). In rhizobia, effectors delivered via the T3SS are thought to assist in the suppression of host immunity, to determine host specificity, and to play a role in nodulation (Okazaki et al., 2013, 2016; Gourion et al., 2015). In the case of PGPR, Rainey (1999) described a T3SS for the PGPR *Pseudomonas fluorescens* SBW25 and since then the T3SS of other fluorescent Pseudomonads was studied (Preston et al., 2001; Rezzonico et al., 2005; Mavrodi et al., 2011). Despite the discovery of T3SSs in the genomes of a number of root-associated *Pseudomonas* spp. (Loper et al., 2012; Berendsen et al., 2015), the role of this secretion machinery and the effectors it delivers remained elusive (Zamioudis and Pieterse, 2012).

Since the beginning of 1990s, three *Pseudomonas* spp. strains have been extensively studied as ISR-inducing PGPR in different plant hosts: *Pseudomonas putida* WCS358, *P. fluorescens* WCS374 and *P. fluorescens* WCS417 (Pieterse et al., 2014). Although WCS358, WCS374, and WCS417 are all capable of eliciting ISR, they show host specificity in terms of their ability to induce ISR in different plant species. For instance, in radish, WCS374 and WCS417 are potent elicitors of ISR, whereas WCS358 is not (Leeman et al., 1996). Conversely, in *Arabidopsis thaliana* (hereafter Arabidopsis), WCS417 and WCS358 are able to trigger ISR, whereas WCS374 is not (Van Wees et al., 1997). Moreover, Arabidopsis possesses natural genetic variation for the ability to express WCS417-mediated ISR, a trait that could be mapped to a single genetic locus in the Arabidopsis genome, indicating that recognition of ISR-inducing rhizobacteria is genetically determined (Ton et al., 1999, 2001). Recently, the genomes of WCS358, WCS374, and WCS417 were described and based on their taxonomy the strains were renamed into *Pseudomonas capeferrum* WCS358, *Pseudomonas defensor* WCS374, and *Pseudomonas simiae* WCS417, respectively (Berendsen et al., 2015). Interestingly, live WCS417 can actively suppress host immune responses in Arabidopsis roots, suggesting a role for *Pseudomonas*-excreted molecules in host immune suppression (Millet et al., 2010; Stringlis et al., 2018).

With the ultimate goal to investigate the role of T3SSs and effectors of WCS374 and WCS417 in beneficial host-microbe interactions (Berendsen et al., 2015), we mapped the T3SS components of WCS374 and WCS417 and found that WCS374 and WCS417 possess T3SS with high similarity to T3SS of other beneficial rhizobacteria. We also found that these rhizobacteria contain sequences of novel effectors in their genomes, which do not elicit hypersensitive response (HR) in tobacco.

## Materials and Methods

### Bioinformatic Analyses

For the bioinformatics analyses, we used the genomes of *P. simiae* WCS417 and *P. defensor* WCS374 described previously (Berendsen et al., 2015). To locate the T3SS gene clusters, we performed BlastP search in the predicted proteome of WCS417 and WCS374, using protein sequences of T3SS conserved components previously identified in the T3SS gene cluster of *Pseudomonas aeruginosa* (Yahr and Wolfgang, 2006). To identify the functions of T3SS components we searched in National Center of Biotechnology Information (NCBI) database for protein sequences with the highest similarity. Alignments and phylogenetic trees were created with CLC main Workbench 6.9 (CLCbio, Aarhus, Denmark) using the neighbor joining algorithm and 1000 bootstrap replicates. T3SS cluster alignments and comparison was performed with progressive MAUVE 2.4.0 (Darling et al., 2010).

### Collection of Root Exudates

*Arabidopsis thaliana* accession Col-0 seeds were surface sterilized and sown on square plates (120 × 120 × 17 mm) containing agar-solidified 1× Murashige and Skoog (MS) medium supplemented with 0.5% sucrose (Murashige and Skoog, 1962). After 2 days of stratification at 4°C, the square plates were positioned vertically and transferred to a growth chamber (22°C; 10 h light: 14 h dark; light intensity: 100 μmol m^–2^ s^–1^). Five days after germination, seedlings were transferred to 6-well plates containing 1× MS supplemented with 0.5% sucrose and kept growing for 7 days. When 12 days old, plants were removed and the remaining growth medium containing the root exudates was filtered (0.22 μM) and stored at −20°C.

### Cultivation of Bacteria and Leaf Infiltration Assay

*Pseudomonas simiae* WCS417 and *P. defensor* WCS374 were cultured at 28°C on King’s medium B (KB; King et al., 1954) agar plates supplemented with 50 μg ml^–1^ of rifampicin (Glandorf et al., 1992). After 24 h of growth, cells were collected in 10 mM MgSO_4_, washed twice by centrifugation for 5 min at 5000 *g* and finally resuspended in 10 mM MgSO_4_. Next, overnight bacterial cultures were initiated with a starting density of OD_600_ = 0.5 (5 × 10^8^ colony-forming units (cfu) per mL) in MS with root exudates (1:1), MS without root exudates (1:1), MgSO_4_ with root exudates (1:1) and MgSO_4_ without root exudates (1:1). The next day, growth of bacteria in these substrates was determined and bacterial densities were adjusted to OD_600_ = 0.1 and 1, after resuspension in 10 mM MgSO_4_. Subsequently, bacterial suspensions were infiltrated in leaves of 5-week-old *Nicotiana tabacum* and *Nicotiana benthamiana* plants using a 1-ml syringe without a needle. Leaves were visually checked for symptoms of an hypersensitive response (HR) 2 days after infiltration. As a positive control for HR induction, tobacco leaves were infiltrated with *P. syringae* pv. *tomato* DC3000.

## Results

### Characterization of T3SS Components in Bacterial Genomes

**FIGURE 1:**
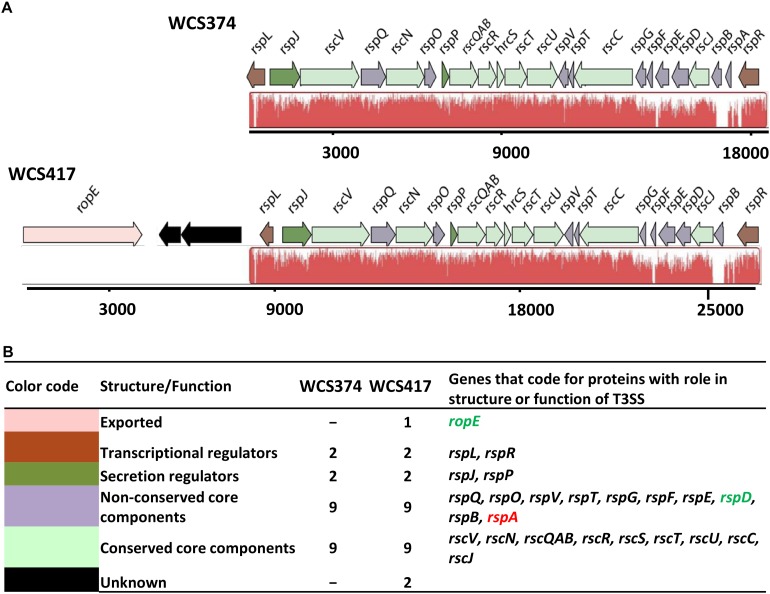
Genetic organization of the T3SS gene clusters of WCS347 and WCS417. **(A)** Orientation and organization of the genes in the T3SS clusters recognized in the genomic sequence of *P. defensor* WCS374 (WCS374) and *P. simiae* WCS417 (WCS417). The red color below the genes indicate regions similar between the two clusters, and the height of the peaks the degree of similarity. **(B)** Each gene in the cluster in panel **(A)** is given a color based on the role its encoding protein has either in the structure or the function of the T3SS apparatus. The color code, the putative role of the protein encoded by each gene, number of genes encoding for proteins with specific role and their names are presented. Green letters indicate gene presence only in the WCS417 cluster, while red letters indicate gene presence only in the WCS374 cluster. The numbers below the gene clusters are indicative of the length of the cluster.

To identify components of the T3SS in the genome sequences of *P. simiae* WCS417 and *P. defensor* WCS374 we performed a BlastP search for homology to genes encoding protein sequences of the canonical T3SS cluster of *P. aeruginosa* (Yahr and Wolfgang, 2006). The search yielded genes corresponding to T3SS components for WCS417 and WCS374 (Berendsen et al., 2015). For the naming of the detected T3SS genes, we followed the nomenclature proposed by Preston et al. (2001) for *P. fluorescens* SBW25 T3SS genes and proteins. In this nomenclature system, genes involved in the structure and regulation of the T3SS are named *rsp* (rhizosphere-expressed secretion protein) or *rsc* (rsp conserved), whereas type III effector genes are named *rop* (rhizosphere-expressed outer protein) (Figure 1A). The genes coding for components of the T3SS in WCS417 and WCS374 reside in a 26-kb and 18-kb region, respectively (Figure 1A) and they exhibit strong similarity in arrangement and orientation. Protein blast searches revealed that the genes of the WCS374 and WCS417 T3SS clusters encode proteins that are highly similar to components of T3SS systems described in other beneficial bacteria (Supplementary Tables S1, S2). As shown in Figure 1, the T3SS gene clusters of WCS374 and WCS417 contain two regulatory proteins that are important for the expression of T3SS associated genes, *rspL* and *rspR* (Preston et al., 2001; Jackson et al., 2005), and nine genes representing core structural components of T3SS systems, *rscV*, *rscN*, *rcsQAB*, *rscR*, *rscS*, *rscT*, *rscU*, *rscC*, and *rscJ* (Tampakaki et al., 2004). In addition to the regulatory and structural T3SS genes, the WCS417 gene cluster contains a gene that shares 79% identity to *ropE*, which codes for a putative effector protein in *P. fluorescens* SBW25 and has similarity with the AvrE effector of the bacterial pathogen *P. syringae* pv. *tomato* DC3000 (Preston et al., 2001; Mudgett, 2005). The WCS374 T3SS gene cluster does not contain this putative effector gene. Comparison of T3SS components of WCS374 and WCS417 gene clusters at protein level using BlastP and comparing components with the same function suggests that most of their components share a degree of similarity ranging from 30 to 97% (Supplementary Table S3).

**FIGURE 2:**
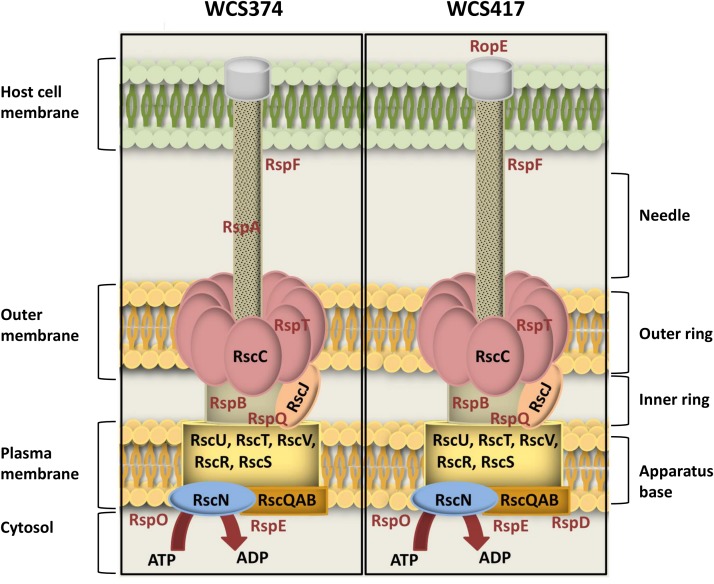
Schematic overview of T3SS-injection machineries of WCS347 and WCS417. Conserved and non-conserved components of the T3SS-body were identified by BlastP and the encoded proteins are placed as parts of the T3SS injectisome, based on their *P. syringae* homologs (Galán et al., 2014). On the right of the figure the basic parts of T3SS body are indicated (apparatus base, inner ring, outer ring and needle). On the left, the cell compartments where the T3SS is spanning from including the bacterial cytosol, plasma membrane and outer membrane and the cell membrane of the host. Conserved components are indicated with black letters, and non-conserved with red letters.

A number of methodologies including microscopy, crystallography and modeling have allowed the visualization of the structure of the T3SS in high resolution (Cornelis, 2010; Worrall et al., 2011). In Figure 2, we placed the proteins encoded by the identified conserved T3SS components of WCS374 and WCS417 as part of the T3SS injectisome, based on their homology with characterized components of this bacterial apparatus in *P. syringae* (Galán et al., 2014). It becomes apparent that the conserved components responsible for the outer ring structure (RscC), inner ring structure (RscJ) and the base of the T3SS apparatus (RscR, RscS, RscU, RscT, RscV) are present in both WCS374 and WCS417. Other conserved components such as the cytosolic ATPase (RscN) and sorting platform component (RscQAB), which are responsible for substrate recognition and initial formation of the apparatus, are also present in both T3SS gene clusters. The only differences between WCS374 and WCS417 are the presence of genes coding for the putative effector RopE and the cytoplasmic protein RspD in WCS417, and the needle filament component RspA (Galán et al., 2014), which is present in WCS374, but not in WCS417. In neither WCS417 nor WCS374, components involved in effector translocation such as rspZ were detected. Other non-conserved genes with a role in T3SS apparatus that could be identified in both bacterial T3SS clusters are *rspF*, *rspT*, *rspB*, *rspQ*, *rspO*, and *rspE*. RspB is a component of the inner rod that connects the needle with the apparatus base, while RspQ is a component of the inner ring of the base. The function of RspO is uknown but is expected to function in the cytoplasm, together with RspE that functions in linking ATP to RscQAB. Finally, RspT interacts with RscC in the formation of the outer ring structure and RspF is involved in pore formation in the cell membrane of the host (Tampakaki et al., 2010; Galán et al., 2014).

**FIGURE 3:**
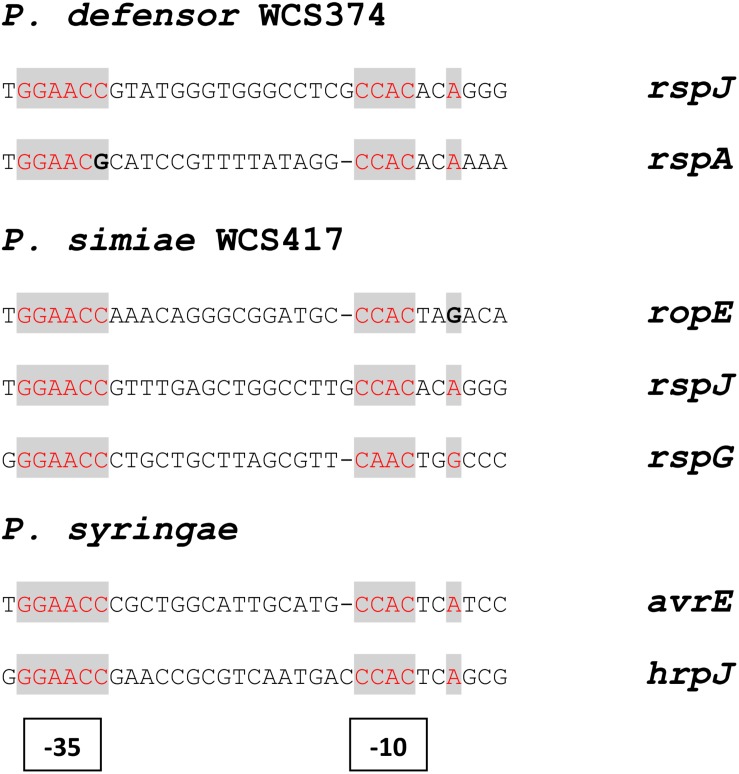
Structure and position of putative hrp boxes identified in WCS374 and WCS417 gene clusters. Putative RspL- binding sites in genes present in the T3SS gene clusters of WCS417 and WCS374 found after manual examination in the promoter region upstream of the indicated genes. Conserved nucleotides are indicated with red letters and divergent nucleotides with bold letters. The box motif upstream of *ropE*, similarly to *avrE*, have a shorter spacer region of 15 nucleotides between the –35 and –10 sites.

Both WCS374 and WCS417 possess a *rspL* and a *rspR* gene in their T3SS gene cluster. In *P. fluorescens* SW25, RspL and RspR have a regulatory role in the expression of other genes present in a T3SS cluster (Preston et al., 2001). In the T3SS gene cluster of the pathogen *P. syringae*, comprised of the *hrp/hrc* genes, the RspR homolog HrpR and HrpS (no homolog found in WCS417 and WCS374) activate the expression of the *rspL* homolog *hrpL*, after which HrpL activates the expression of the *hrp*, *hrc* genes and the effector-encoding *avr* genes by binding to a conserved promoter motif, the “hrp box” (GGAACC-N15/16-CCACNNA), present in the promoter region of these genes (Tampakaki et al., 2010). The presence of this motif is a good indication for regulation of T3SS-related genes within a genome (Greenberg and Vinatzer, 2003). Manual examination of the WCS417 and WCS374 T3SS gene clusters revealed the existence of hrp box promoter elements upstream of *ropE*, *rspJ*, and *rspG* in WCS417 and upstream of *rspJ* and *rspA* in WCS374 (Figure 3).

### Phylogenetic Relationship of WCS374 and WCS417 T3SS With Those of Other Beneficial and Pathogenic *Pseudomonas* spp.

**FIGURE 4:**
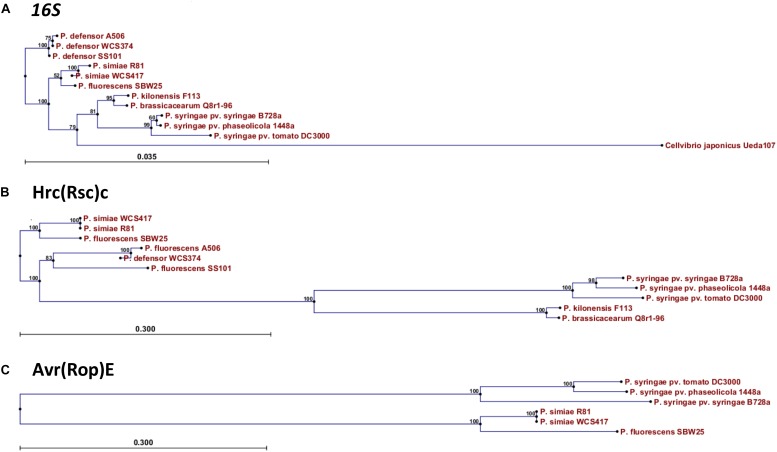
Phylogenetic relationship of WCS417 and WCS374 T3SS components with other beneficial and phytopathogenic *Pseudomonas* spp. Neighbor-joining phylogenetic trees for aligned nucleotide sequence of *16S rRNA* **(A)** and protein sequences of Hrc(Rsc)C **(B)** and Avr(Rop)E **(C)**. In the case of *16S rRNA* the evolutionary distance was calculated with Kimura 80 model, while evolutionary distance between protein sequences was estimated with Jukes-Cantor model. Numbers on nodes are calculated with bootstrap test using 1000 replicates. The length of branches is corresponding to the amount of changes in the time of evolution.

In order to investigate the relationship of WCS358, WCS374, and WCS417 with other *Pseudomonas* spp. strains, the concatenated sequences of the housekeeping genes *16S rRNA*, *gyrB*, *rpoB*, and *rpoD* were compared to those of 107 *Pseudomonas* sp. type strains (Berendsen et al., 2015). On the basis of this analysis WCS358 was placed in the *P. putida* group, whereas WCS374 and WCS417 were placed in the *P. fluorescens* group of the phylogenetic tree of the *Pseudomonas* genus (Mulet et al., 2010). To analyze the phylogenetic relationship of the T3SS gene clusters of WCS374 and WCS417, we first built a phylogenetic tree based on the *16S rRNA* genomic sequence of WCS417 and WCS374 and those of the plant-beneficial *P. fluorescens* group species *simiae* R81, *P. fluorescens* SBW25, *P. defensor* SS101, *P. defensor* A506, *P. kilonensis* F113 [previously *P. brassicacearum* F113; (Almario et al., 2017)], and *P. brassicacearum* Q8r1-96, and the phytopathogenic *P. syringae* group species *P. syringae* pv. *syringae* B728a, *P. syringae* pv. *phaseolicola* 1448a, and *P. syringae* pv. *tomato* DC3000. Neighbor-Joining (NJ) phylogeny was used to construct a tree depicting evolutionary distance between the different species, using the *16S rRNA* sequence from *Cellvibrio japonicus* strain Ueda 107 as an outgroup for our analysis (Figure 4). The resulting NJ phylogenetic tree contained a clade for the outgroup *C. japonicus*, a clade with the pathogenic *P. syringae*, and three clades with plant-beneficial *Pseudomonas* spp., i.e., *P. brassicacearum* and *P. kilonensis*, *P. simiae* and *P. fluorescens*, and *P. defensor*, respectively (Figure 4A). In this analysis, the *P. brassicacearum*/*P. kilonensis* spp. are more closely related to pathogenic *P. syringae* spp. than to the other plant-beneficial *Pseudomonas* spp., confirming previous findings (Berendsen et al., 2015).

Next, we performed a similar phylogenetic analysis using the protein sequences of the conserved T3SS component HrcC (RscC in beneficial rhizobacteria) and the conserved effector AvrE (RopE in beneficial rhizobacteria). The NJ phylogenetic tree built with the conserved HrcC sequence revealed that there is a considerable evolutionary distance between the beneficial *Pseudomonas* species related to WCS374 and WCS417 and pathogenic *P. syringae* (Figure 4B). Conversely, the HrcC sequence of the *P. brassicacearum* and *P. kilonensis* is more closely related to those of the phytopathogenic *P. syringae* (Figure 4B). The protein sequences of homologs of RopE could only be retrieved for the beneficial *Pseudomonas* spp. *P. fluorescens* SW25 and *P. simiae* WCS417 and R81. However, the evolutionary distance between the RopE sequences of the beneficial and pathogenic *Pseudomonas* spp. was similarly large as that for HrcC/RscC (Figure 4C).

**FIGURE 5:**
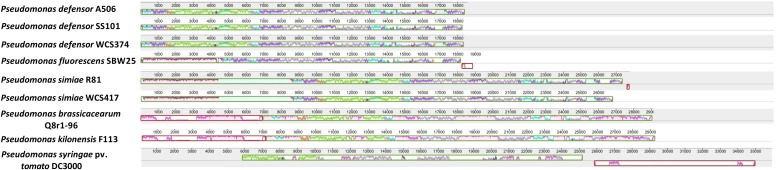
Similarity of WCS417 and WCS374 T3SS gene clusters with other beneficial and phytopathogenic *Pseudomonas* spp. Gene clusters were identified in available sequenced genomes of beneficial and phytopathogenic *Pseudomonas* spp. Progressive Mauve was used to align the clusters and find regions exhibiting high similarity. Similar colors correspond to similar genomic regions in a cluster. The height of peaks in each cluster depicts the degree of similarity in regions present in T3SS clusters of different *Pseudomonas* spp.

Next, we aligned the genome sequences of the whole T3SS gene clusters of the beneficial *Pseudomonas* spp. strains and that of the pathogen *P. syringae* pv. *tomato* DC3000 using Progressive Mauve (Figure 5). The color coding in this alignment indicates similarity between T3SS components of the clusters and the height of the peaks the level of similarity. Figure 5 shows that the T3SS gene clusters of the beneficial *Pseudomonas* spp. strains display a high degree of similarity, the degree of which is in line with the phylogenetic clustering displayed in Figure 4. The T3SS gene clusters of *P. defensor* strains WCS374, A506, and SS101 are highly similar. The same holds true for the T3SS gene clusters of the *P. simiae* strains WCS417 and R81. The T3SS gene cluster of *P. fluorescens* SBW25 differentiates from those of the *P. defensor* and *P. simiae* strains, but this is largely due to the presence of *ropE* at the beginning of the cluster (absent in the *P. defensor* strains), and the absence of a number of components that are present in the *P. defensor* and *P. simiae* strains (green region). Like *P. fluorescens* SBW25, the two *P. simiae* strains (R81 and WCS417) contain the genomic region encoding *ropE* (purple region located in the first 4000 bp). This genomic region in the T3SS clusters of WCS417 and R81 is followed by a genomic region of ∼4000 bp that is not present in the other species (light blue region). The remaining part of the T3SS gene cluster of WCS417 and R81 is quite similar to that of the *P. defensor* strains WCS374, SS101 and A506. We included *P. brassicacearum* and *P. kilonensis* strains in this analysis due to their intermediate phylogenetic distance between the *P. defensor* and *P. simiae* strains and the phytopathogenic ones (Berendsen et al., 2015). Only some parts of the T3SS gene clusters found in the *P. kilonensis* F113 and *P. brassicacearum* Q8r1-96 aligned with the other beneficial *Pseudomonas* spp. strains. These clusters contain regions (first 7000 bp, 14500–16000 bp, 18000–20500 bp, 26000–29000 bp) that are not detected in the other beneficial *Pseudomonas* spp. strains. The T3SS gene cluster of *P. syringae* pv. *tomato* DC3000 is significantly longer than those of the beneficial *Pseudomonas* spp. strains and only few genomic regions aligned with the T3SS gene clusters of the beneficial *Pseudomonas* spp. (Figure 5).

### Identification of Putative Effector Proteins in WCS417 and WCS374

From the computational analysis presented above, *ropE* was identified in the WCS417 T3SS gene cluster as a gene encoding a putative secreted effector. However, genes encoding secreted effector proteins are not always in the genomic proximity of the T3SS gene cluster and can be scattered around the genome (Peters et al., 2007). A common method to identify type III effectors in bacterial genomes is by searching for genes that have conserved Hrp(Rsp) “box motifs” in their promoter regions (Supplementary Figure S1). This hrp box is defined by the following sequence: xGGAACx[N_15–16_]CCACxxAG and the space between the motif and the regulated gene varies from 30 to 300 bp (Zwiesler-Vollick et al., 2002). By screening the genomes of WCS374 and WCS417 for such hrp box motifs, we identified 62 and 73 genes encoding putative secreted effector proteins, respectively. In a next step, we analyzed their N-terminal protein sequence for characteristics typical of T3SS-secreted proteins [i.e., abundance of Ser and polar residues, one acidic residue in the first 12 positions, and an aliphatic amino acid in position 3 or 4; (Guttman et al., 2002; Petnicki-Ocwieja et al., 2002)]. This extra step resulted in a final list of 15 putative effectors for WCS374 and 11 putative effectors for WCS417 (Berendsen et al., 2015).

A BlastP search with the protein sequences of these putative effectors revealed that the putative effectors of WCS374 were quite similar with previously identified effectors of *P. defensor* A506 (Loper et al., 2012), while putative effectors of WCS417 displayed high homology with proteins identified in *P. simiae* R81 (Supplementary Tables S4, S5). Among the identified putative effectors are proteins previously characterized as T3SS-secreted effectors such as RopE (Preston et al., 2001), ExoU and HopJ. ExoU is a type III-delivered toxin of *P. aeruginosa* associated with bacterial spreading and lung injury in humans and animals (Finck-Barbancon et al., 1997). HopJ is a T3SS-secreted effector with high degree of conservation among beneficial and pathogenic *Pseudomonas* spp. (Lindeberg et al., 2005; Trantas et al., 2015). However, the majority of the WCS417 and WCS374 effectors show no homology to previously identified effectors.

### WCS374 and WCS417 Do Not Trigger an HR in Tobacco

Previously Preston et al. (2001) tested whether *P. fluorescens* SBW25 could elicit HR upon infiltration in leaves of different plant hosts. To assess whether putative effectors of WCS374 and WCS417 can be recognized by tobacco R genes and induce HR, we infiltrated *N. tabacum* and *N. benthamiana* leaves with bacteria growing in the presence or absence of exudates, aiming to induce the activation of T3SS (Anderson et al., 2014). As shown in Supplementary Figure S2, the beneficial rhizobacteria did not elicit HR symptoms, unlike *P. syringae* pv. *tomato* DC3000 which induced a visible and clear HR in all plants and concentrations tested. From this experiment, it seems that either none of WCS374/WCS417 effectors are recognized by these plants or that the system is not specific for root-inhabiting microbes. Further studies, employing T3SS mutants and heterologous expression systems could demonstrate the functionality of these secretion machineries.

## Discussion

Selected root-inhabiting microbes are known to provide their plant hosts with benefits such as growth promotion, facilitation of nutrient uptake, and biological control of root-infecting pathogens (Zamioudis and Pieterse, 2012). Among these beneficial microbes, specific *Pseudomonas* species are extensively studied for their ability to trigger broad-spectrum ISR (Pieterse et al., 2014). The establishment of this plant defense mechanism requires colonization of plant roots by ISR-triggering microbes in high population densities (Raaijmakers et al., 1995). Additionally, the ability of beneficial *Pseudomonas* spp. to trigger ISR is host specific (Leeman et al., 1996; Berendsen et al., 2015). These characteristics of ISR suggest that specific microbial functions contribute to the suppression of host immunity, allowing the beneficial microbe to colonize its host in a host-microbe genotype specific manner. Since the T3SS-secreted effectors of phytopathogenic bacteria provide such host-selective microbial functions (Alfano and Collmer, 2004), we aimed at investigating the T3SSs of the well-studied ISR-inducers *P. simiae* WCS417 and *P. defensor* WCS374 (Pieterse et al., 2014). The genomic elucidation of these strains by Berendsen et al. (2015) showed that WCS374 and WCS417 contain a T3SS in their genome, whereas WCS358 does not. In the present study, we show that the genomes of WCS417 and WCS374 possess most of the components of a functional T3SS. The T3SS genes of WCS417 and WCS374 reside in clusters of 26 and 18 kb, respectively, and share strong similarities in their organization and orientation (Figure 1). The T3SS gene clusters of these rhizobacteria contain each more than 20 genes, encoding structural and regulatory proteins of T3SS (Supplementary Tables S1, S2). The first T3SS in a beneficial *Pseudomonas* spp. was described for *P. fluorescens* SBW25 (Preston et al., 2001), which was present in a 20-kb genomic region. Since then, several other T3SS gene clusters of saprophytic plant-associated *Pseudomonas* spp. have been identified, all located in a genomic region ranging between 18 and 28 kb (Loper et al., 2012). In phytopathogenic *Pseudomonas* spp., HrpR and HrpS proteins regulate the expression of *hrpL*, encoding alternative sigma factor HrpL, which is required for the activation of genes encoding components of the T3SS (Xiao et al., 1994; Jovanovic et al., 2011). The T3SS clusters of WCS417 and WCS374 both possess the *hrpL* and *hrpR* homologs *rspL* and *rspR*, but, like *P. fluorescens* SBW25 (Preston et al., 2001), lack a homolog of *hspS* (Figure 1). HrpL recognizes a specific hrp box sequence present in the promoter region of the genes it regulates (Tampakaki et al., 2010). Our analysis of the WCS374 and WCS417 genomes revealed the existence of such motifs upstream of genes present in the T3SS clusters of both bacteria, suggesting RspL-dependent regulation of T3SS components (Figure 3), as was already suggested for SBW25 (Jackson et al., 2005) and *P. syringae* (Xiao and Hutcheson, 1994). Most of the components identified in the T3SS clusters in this study correspond to proteins responsible for the formation of intracellular parts of the T3SS-injection machinery as well as for the formation of the ring structures (Figure 2). In other plant-beneficial *Pseudomonas* spp., such as *P. brassicacearum* Q8r1-96 and *P. fluorescens* 2P24, additional proteins responsible for pilus and pore formation were identified in the T3SS cluster (Mavrodi et al., 2011; Liu et al., 2015). Proteins responsible for needle formation were detected only in the case of WCS374 (RspA) but not in WCS417, while no proteins associated with effector translocation could be found in the T3SS clusters of WCS374 and WCS417 (Figure 2). All these findings together suggest that both WCS417 and WCS374 have T3SS gene clusters containing all components for its activation and depending on the bacterium some components for the formation of an apparatus that could potentially deliver effector molecules.

Phylogenetic analysis using the highly conserved T3SS protein sequence RscC revealed that the T3SS of WCS417 and WCS374 are evolutionary different from those of the plant-beneficial bacterium *P. brassicacearum* Q8r1-96 (Mavrodi et al., 2011) and phytopathogenic *Pseudomonas* species (Figure 4). Neighbor joining phylogeny of the conserved T3SS component (RscC) and RopE effector protein placed WCS417 in the same clade with its close relative *P. simiae* R81 and *P. fluorescens* SBW25, while RscC phylogeny placed WCS374 in a different clade together with *P. defensor* A506 and *P. defensor* SS101 (Figure 4). These phylogenetic relationships are in line with those obtained by Berendsen et al. (2015) who aligned the concatenated sequences of the housekeeping genes *16S rRNA*, *gyrB*, *rpoB*, *rpoD*. Moreover, alignment of whole T3SS clusters with Progressive Mauve (Figure 5) further supported the phylogenetic observations. In that case not only one gene but the whole cluster was compared in terms of homology and organization and it became apparent that WCS417 possesses a cluster representative of *P. simiae* strains, while WCS374 a cluster representative of *P. defensor* strains. On the other hand, the phylogenetically distant *P. brassicacearum* and *kilonensis* strains (Q8r1-96 and F113, respectively) have larger clusters resembling more the T3SS of *P. syringae* pv. *tomato* DC3000 than those of the other beneficial *Pseudomonas* spp.

Previously, we looked whether putative effectors could be identified in the genomes of WCS417 and WCS374, following the workflow presented in Supplementary Figure S1. This methodology allowed us to predict the existence of 15 and 11 putative effectors in WCS374 and WCS417, respectively (Berendsen et al., 2015). In other beneficial *Pseudomonas* spp., the number of putative effectors following prediction ranged between 4 and 16 (Loper et al., 2012). Most of the proteins identified in this study are novel and could not be linked to known effector families (Supplementary Tables S4, S5). One exception is the prediction of RopE in WCS417, which resides in the T3SS gene cluster of WCS417 next to rspL transcriptional regulator and shares similarity with T3SS secreted protein AvrE from *P. syringae* (Preston et al., 2001). AvrE proteins are major virulence factors in plant pathogenic bacteria, and they have been found to target salicylic-acid mediated defense responses in plants, facilitating infection of the host (DebRoy et al., 2004; Ham et al., 2009). Moreover, proteins ExoU and HopJ were found in both WCS417 and WCS374 and are known to be T3SS-secreted in other systems (Finck-Barbancon et al., 1997; Lindeberg et al., 2005). The activity of T3SS and the effector molecules delivered via this secretion machinery could have various roles either in microbe-microbe or plant-microbe interactions. Both pathogenic and beneficial microbes need to modulate host defense responses to establish an interaction with their host (Zamioudis and Pieterse, 2012). In PGPR *P. fluorescens* SBW25, expression of conserved T3SS components like *rscC* gene was observed in the rhizosphere of sugar beet plants (Rainey, 1999). Nodulation in some legume species can happen independently of rhizobial Nod-factors, but through the activity of T3SS (Okazaki et al., 2013, 2016). The role of T3SS is also demonstrated in mycorrhization, since T3SS-harboring bacteria (among them *P. fluorescens* species) are enriched in the rhizospheres of plants forming symbiosis either with ectomycorrhizal fungus (EMF) *Laccaria proxima* or arbuscular mycorrhizal fungi (AMF) (Warmink and Van Elsas, 2008; Viollet et al., 2011). More recently, Viollet et al. (2017) demonstrated that a T3SS mutant of *P. fluorescens* C7R12 could not promote root colonization of *Medicago truncatula* by AMF. Additionally, effectors of PGPR *P. brassicacearum* Q8r1-96 can suppress plant immune response when injected in tobacco leaves (Mavrodi et al., 2011). The presence of T3SS in rhizosphere-inhabiting bacteria suggests their possible involvement in local suppression of root immune responses therewith facilitating root colonization by the PGPR or its interacting partners, like AMF.

Finally, we aimed to test whether both rhizobacteria could trigger local immune responses in leaves of tobacco following their infiltration. HR is an indication of activated plant resistance locally at the site of the infection, due to the recognition of effector molecules delivered by bacterial pathogens (Mur et al., 2008). However, no visible HR symptoms were observed in leaves of both *N. benthamiana* and *N. tabacum* (Supplementary Figure S2). Similar observations were made in other beneficial Pseudomonas strains (Preston et al., 2001; Liu et al., 2015). The explanation in this case could be that either the system of study is not optimal, since we infiltrated leaves with rhizosphere-specific microbes and expression of T3SS is lower, or that the secreted molecules are not recognized by the hosts tested (Preston et al., 2001). Additionally, the role of T3SS present in PGPR bacteria could have a role before plant colonization, when bacteria need to compete with other bacterial or fungal species for the same niches (Rezzonico et al., 2005) or in suppressing local root immune responses (Millet et al., 2010; Mavrodi et al., 2011; Stringlis et al., 2018). Elucidation of the role of T3SS and putative effector molecules found in WCS417 and WCS374 could be achieved either by generating bacteria defective in T3SS activation or by employing heterologous systems for effector delivery (Mavrodi et al., 2011; Liu et al., 2015).

To sum up, our data confirm the presence of T3SS gene clusters in the beneficial PGPR rhizobacteria *P. simiae* WCS417 and *P. defensor* WCS374. Moreover, components of the T3SS gene clusters from WCS417 and WCS374 exhibit high phylogenetic similarity with other beneficial rhizobacteria but are distinct compared to phytopathogenic *P. syringae*. Identification of novel effector molecules was possible in WCS417 and WCS374, but further study could explain the role of T3SS clusters and T3SS-secreted proteins in these beneficial rhizobacteria and their involvement in ISR-host specificity and rhizosphere competence.

## Data Availability

All datasets generated for this study are included in the manuscript and/or the Supplementary Files.

## Author Contributions

IS performed the experiments and analyzed the data. IS and CP wrote the manuscript. All authors conceived and designed the experiments and read and approved the manuscript.

## Conflict of Interest Statement

The authors declare that the research was conducted in the absence of any commercial or financial relationships that could be construed as a potential conflict of interest.
