# Supplementary material for: Type III Secretion System of Beneficial Rhizobacteria Pseudomonas simiae WCS417 and Pseudomonas defensor WCS374
Source: Front Microbiol. 2019 Jul 16;10:1631. doi: 10.3389/fmicb.2019.01631 (PMC6647874; doi:10.3389/fmicb.2019.01631)
Supplement: Supplementary file 1 [file Data_Sheet_1.PDF]

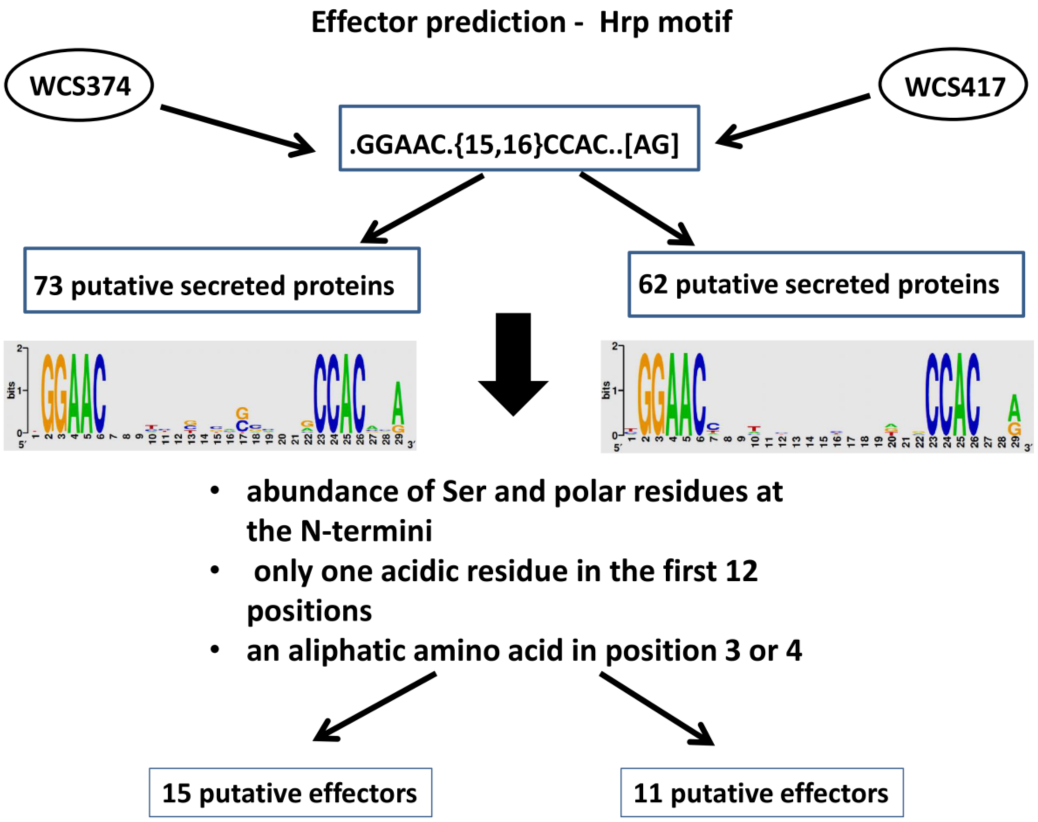

Supplementary Figure S1. Workflow for the identification of putative effectors in the genome of WCS417 and WCS374. Genomes of WCS374 and WCS417 were searched for the presence of hrp box motif in gene promoter regions. The candidates found by the hrp box search were further screened for characteristics of their protein, typical of T3SS-effectors. These characteristics were searched in the first 50 aa of the N-terminal region.

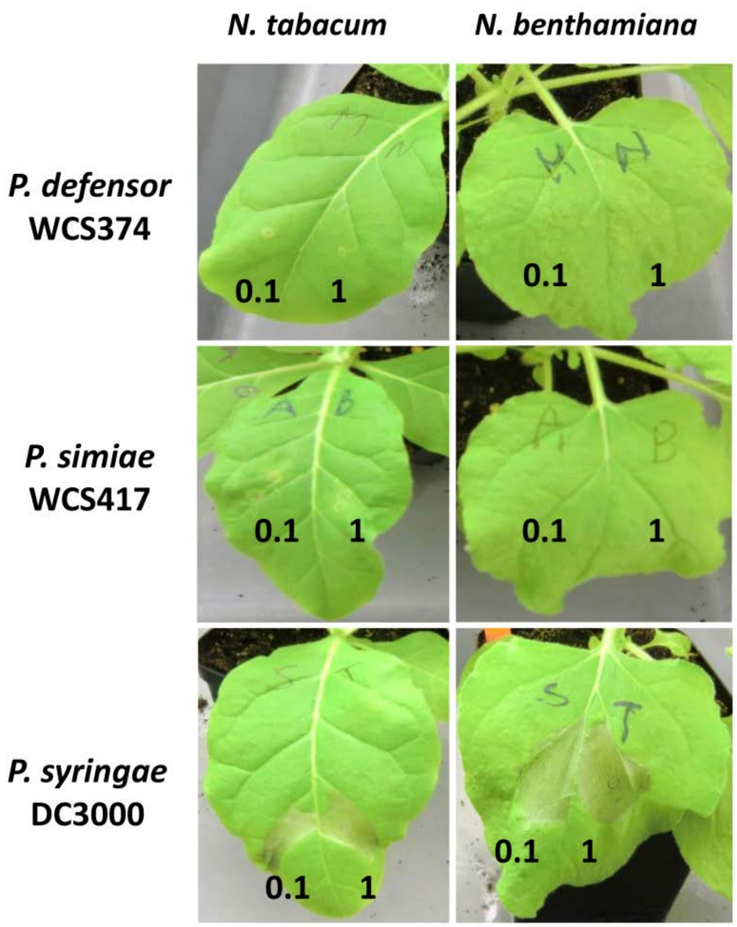

Supplementary Figure S2. WCS374 and WCS417 are unable to elicit HR symptoms in tobacco leaves. Leaves of *N. tabacum* (left side) and *N. benthamiana* (right side) were infiltrated with a bacterial suspension at OD_{600}: 0.1 and 1, using a 1-ml blunt-ended syringe. Bacteria were grown in MS with exudates, MS without exudates, $MgSO_4$ with exudates and $MgSO_4$. In the pictures, infiltrations are shown which were performed with bacteria grown in MS with exudates (all other growth media gave similar results). *P. syringae* DC3000 was used as a positive control. Photographs of symptoms were taken at 2 d after infiltration.
